# Sampling clustering based on multi-view attribute structural relations

**DOI:** 10.1371/journal.pone.0297989

**Published:** 2024-05-23

**Authors:** Guoyang Tang, Xueyi Zhao, Yanyun Fu, Xiaolin Ning

**Affiliations:** 1 College of Information Science and Engineering, Xinjiang University, Urumqi, Xinjiang, China; 2 China Electronics Technology Electronics Technology Academy Technology Group Co., Ltd., Beijing, China; 3 XinJiang Lianhai INA-INT Information Technology Co., Ltd., Urumqi, China; 4 Xinjiang Social Security Risk Intelligent Perception Engineering Research Center, Urumqi, China; 5 Beijing Academy of Science and Technology, Beijing, China; Zhejiang University of Technology, CHINA

## Abstract

In light of the exponential growth in information volume, the significance of graph data has intensified. Graph clustering plays a pivotal role in graph data processing by jointly modeling the graph structure and node attributes. Notably, the practical significance of multi-view graph clustering is heightened due to the presence of diverse relationships within real-world graph data. Nonetheless, prevailing graph clustering techniques, predominantly grounded in deep learning neural networks, face challenges in effectively handling multi-view graph data. These challenges include the incapability to concurrently explore the relationships between multiple view structures and node attributes, as well as difficulties in processing multi-view graph data with varying features. To tackle these issues, this research proposes a straightforward yet effective multi-view graph clustering approach known as SLMGC. This approach uses graph filtering to filter noise, reduces computational complexity by extracting samples based on node importance, enhances clustering representations through graph contrastive regularization, and achieves the final clustering outcomes using a self-training clustering algorithm. Notably, unlike neural network algorithms, this approach avoids the need for intricate parameter settings. Comprehensive experiments validate the supremacy of the SLMGC approach in multi-view graph clustering endeavors when contrasted with prevailing deep neural network techniques.

## 1 Introduction

Graph clustering involves partitioning a graph into several disjoint clusters of nodes [1]. Multi-view clustering, building upon graph clustering, leverages richer graph information by seeking consistent clustering results through multiple view relationships. Graph data techniques find widespread applications in various practical situations, such as group segmentation [2], social graphs [3], sentiment analysis [4–6] and the traffic classification [7, 8].

Multi-view graph clustering has evolved from single-view clustering, with LINE [9] and GAE [10] being two representative algorithms in this context. LINE aims to map nodes in a graph to a low-dimensional space, making adjacent nodes closer in the embedding space. It achieves this by maximizing the similarity of positive samples and minimizing the similarity of negative samples. GAE employs the idea of autoencoders to learn node embeddings while preserving the structural information of the graph. The encoder part of the autoencoder maps nodes to a low-dimensional space, and the decoder attempts to reconstruct the original graph from this low-dimensional representation. However, real-world graph data often involves more relationships, and single-view clustering may not leverage these additional connections to explore deeper information.

Early multi-view clustering techniques can be broadly categorized into two types. One approach involves obtaining a consensus graph from multiple views and applying a single-view algorithm to it, such as RMSC, PwMC, and SwMC [11–13]. Another approach utilizes graph embedding to obtain compact representations of nodes from multi-view data and then applies classical clustering algorithms, such as PMNE, mvn2vec, and SMNE [14–16]. However, these algorithms fail to simultaneously leverage both node attributes and graph relationships.

In recent years, inspired by Graph Convolutional Networks (GCN) [17], two types of multi-view attribute graph clustering algorithms have emerged. One2Multi (O2MA) [18] posits that there exists one view containing the most information among multiple views. Therefore, O2MA employs a graph autoencoder based on one view to embed nodes and reconstruct multiple views. However, this approach fails to fully leverage the structural relationships between different views. MAGCN [19] primarily deals with the clustering of multiple node attribute graphs under a single structural relationship. Clearly, they are not effective in handling graph data with multiple node attributes and multiple view structures.

The graph learning module can effectively address the issue of simultaneously leveraging node attributes and structural relationships. MAGC [20], MCGC [21], and HMvC [22] employ graph learning modules to address the computationally complex nature of neural network parameters. However, their computational efficiency still needs improvement for large datasets, and the use of traditional clustering methods during final clustering results in insufficient stability.

Existing multi-view clustering algorithms have the following shortcomings:

1. Real-world graph data often contains noise or missing values. Deep neural networks heavily rely on the quality of raw graph data, and they lack interpretability.
2. Using neural network methods for multi-view clustering with large samples is computationally complex, time-consuming, and memory-intensive.
3. In the final clustering stage, many existing multi-view clustering methods utilize traditional clustering methods such as k-means. However, these clustering methods exhibit high randomness, leading to significant variations in results with each computation.

**Fig 1:**
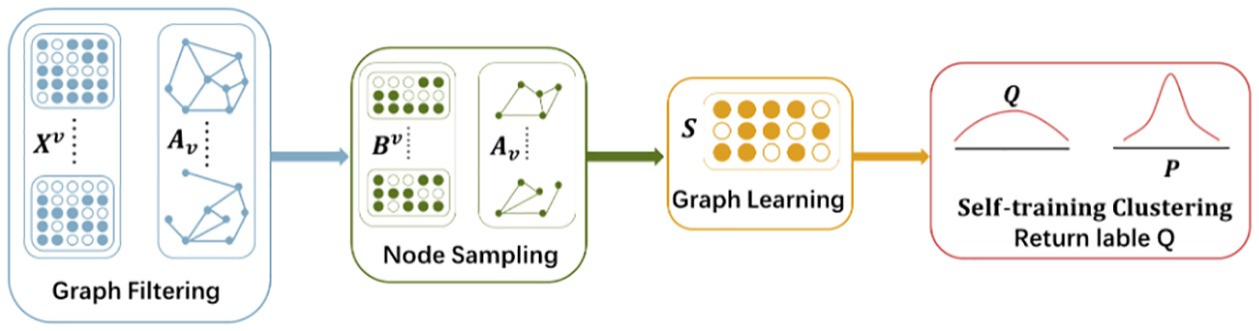
Structure of SLMGC.

In an effort to overcome the mentioned constraints, we introduce a novel approach for multi-view clustering, referred to as SLMGC. The complete structure of SLMGC is illustrated in Fig 1. In this paper, we provide a comprehensive algorithmic explanation of the clustering algorithm and conduct a thorough analysis of its individual modules. The primary contributions of this investigation can be outlined as follows:

1. Graph filtering is employed as a replacement for Graph Convolutional Neural Networks (GCN) to obtain node embeddings from the feature matrix. This approach mitigates the impact of noise in the initial data on the eventual clustering outcomes.
2. A sampling algorithm is utilized to select a batch of nodes, which effectively reduces computational costs and diminishes the impact of outliers on the clustering outcomes.
3. A contrastive loss is used as a regularization term, enabling the utilization of both structural information and features from various views to construct the consensus graph. Moreover, a self-training clustering algorithm is designed to enhance the stability of the final clustering results and reduce clustering bias commonly observed in traditional approaches.

## 2 Relevant concepts and definitions

Multi-view data refers to data composed of multiple relationships represented in multiple views, where each node in the graph corresponds to a sample point. The edges between node pairs in each view represent the relationships between those nodes in that specific view. The relationships in each view are represented by adjacency matrices, and each node is associated with its own attributes or features, represented by vectors. The feature vectors of all nodes collectively form the feature matrix for that particular view.

### 2.1 Multi-view graph data

Let $G=\{V,E_{1},\ldots ,E_{V},X^{1},\ldots ,X^{V}\}$ denote the multi-view data, where the set of N nodes is denoted as V, *e*_*ij*_ ∈ *E*_*v*_ indicates the presence of an edge among node i and node j in the v-th view and belongs to the set $E_{v}.X^{v}={\left\{x_{1}^{v},\ldots ,x_{N}^{v}\right\}}^{\text{T}}\in R^{N\times d_{v}}$ represents the feature matrix of the v-th view, comprising N attribute vectors of length *d*_*v*_.

### 2.2 Laplacian matrix

The relationship structure of each view, i.e., the presence of edges, can be represented using the adjacency matrix ${\left\{\tilde{A_{v}}\right\}}_{v=1}^{V}$, where $\tilde{A_{v}}=\left\{\tilde{a_{ij}^{v}}\right\}\in R^{N\times N}$. If there exists an edge between node i and node j, $\tilde{a_{lj}^{v}}=1$, otherwise, $\tilde{a_{lj}^{v}}=0$. *D*_*v*_ is the degree matrix of the adjacency matrix $\tilde{A_{v}}$. Considering the self-expressive property of nodes in the graph, where a node can be linearly represented by its neighboring nodes, we introduce the normalized adjacency matrix denoted as $A_{v}={D_{v}}^{-\frac{1}{2}}\left(\tilde{A_{v}}+I\right){D_{v}}^{-\frac{1}{2}}$, its associated graph Laplacian matrix represented as *L*_*v*_ = *I* − *A*_*v*_, where I is the identity matrix.

## 3 Algorithm

### 3.1 Graph filtering

In real-world graph data, neighboring nodes often exhibit certain similarities in their features. Therefore, we introduce the concept of graph filtering [23]. To facilitate the subsequent discussions, we first focus on the single-view scenario. The feature matrix *X* ∈ *R*^*N*×*d*^ can be viewed as a collection of N graph signal vectors, denoted as *f*. The Laplacian matrix can be decomposed as ***L*** = ***U*Λ*U***^−1^, where **Λ** = *diag*(λ_1_, ⋯, λ_*N*_) represents the increasing eigenvalues, and ***U*** = [*u*_1_, ⋯, *u*_*N*_] corresponds to the related orthogonal eigenvectors. The graph filter can be expressed as *G*_*f*_ = *Up*(Λ)*U*^−1^ ∈ *R*^*N*×*N*^, where *p*(Λ) = diag(*p*(λ_1_), ⋯, *p*(λ_*N*_)) denotes the frequency-response function [24]. The graph filtering operation can be defined as the multiplication between the graph signal and the graph filter:

$$\begin{array}{c} \bar{f}=G_{f}f, \end{array}$$ (1)

The filtered graph signal is denoted as $\bar{f}$.

To facilitate clustering, we desire nodes within the same cluster to possess similar feature values across all dimensions. Based on this assumption about clusters, we consider that nodes closer in distance are more likely to belong to the same cluster. However, directly applying graph filters to the feature matrix may not fully exploit the graph’s structural information, as first-order graph filters only smooth the neighboring nodes within one hop. Therefore, we consider the use of k-th order graph filtering to capture longer-distance graph structural information. We specify the k-th order graph filtering as follows:

$$\begin{array}{c} \bar{X}={\left(I-\frac{1}{2}L\right)}^{k}X, \end{array}$$ (2)

Where $\bar{X}$ represents the feature matrix after filtering.

### 3.2 Graph learning

Considering that real-world graph data often contain noise and missing values, directly applying spectral clustering to $\bar{X}$ may not yield satisfactory clustering results. Utilizing the self-expressive characteristic of graph data [25], where each node can be expressed as a linear combination of other nodes, we learn a similarity graph Z from $\bar{X}$. The coefficients of node combinations represent the relationships between nodes, which can also be viewed as distances in classical clustering tasks. The objective function for single-view is as follows:

$$\begin{array}{c} min\|{\bar{X}}^{\text{T}}-{\bar{X}}^{\text{T}}{Z\|}_{F}^{2}+\alpha {\left\|Z\right\|}_{F}^{2}, \end{array}$$ (3)

Where *α* > 0is a weight parameter, *Z* ∈ *R*^*N*×*N*^ is the consensus graph matrix. The primary component represents the reconstruction loss, and the secondary component represents the regularization term.

To handle multi-view data, we apply graph filtering to each view’s feature matrix, resulting in ${\bar{X}}^{v}$. Subsequently, we extend Eq 3 by introducing a weight parameter for each view to determine their respective importance. Ultimately, we obtain the consensus graph for all views as follows:

$$\begin{array}{c} \underset{Z,\lambda^{v}}{\text{min}}\;\sum_{v=1}^{V}\;\lambda^{v}\left(\right\|{\bar{X}}^{v\text{T}}-{\bar{X}}^{v\text{T}}{Z\|}_{F}^{2}+{\alpha \|Z\|}_{F}^{2})+\sum_{v=1}^{V}{\left(\lambda^{v}\right)}^{\omega }, \end{array}$$ (4)

λ^*v*^ represents the coefficient value for the v-th view, and *ω* < 0 is the smoothing factor.

### 3.3 Node sampling

In the case of large datasets with a considerable number of nodes, direct spectral clustering on the obtained consensus graph Z could lead to long computation times and high memory usage. Additionally, considering the impact of outliers may result in a decrease in clustering accuracy. To address these issues, we refrain from using the previously filtered $\bar{X}$ and instead opt to extract *m*(*m* < *N*) key sample points that hold significance within the graph [26].

Classic sampling algorithms assume equal weights for each point, but in graph data, different nodes hold varying levels of importance. Inspired by word sampling techniques in NLP [27], we perform sampling based on node importance, where nodes with a higher number of edges in each view are considered to be more important. We define $q\left(i\right)=\sum_{v=1}^{V}\sum_{j\in V}\tilde{A_{ij}^{v}}$ as the function for measuring importance. The likelihood of each node i being the first sample in the sampling set M is given by:

$$\begin{array}{c} p_{i}=\frac{q{\left(i\right)}^{\gamma }}{\sum_{j\in V}\left(q{\left(j\right)}^{\gamma }\right)}, \end{array}$$ (5)

Where *γ* > 0. Subsequently, we employ a non-replacement sampling algorithm to select the remaining m-1 samples. Specifically, each remaining node i is selected as the next sample with a probability of *p*_*i*_/Σ_*j*∉*M*_ *p*_*j*_, until m nodes have been sampled.

Now, we construct the feature matrix *B* = {*b*_1_, ⋯, *b*_*m*_}^T^ ∈ *R*^*m*×*d*^ using the sampled node feature vectors, and B is a part of $\bar{X}$. Ultimately, we acquire a reduced consensus graph matrix *S* ∈ *R*^*m*×*N*^ through the learning process, which represents the similarity between the m sampled nodes and all N nodes. As a result, we can reformulate Eq 4 as follows:

$$\begin{array}{c} \underset{S,\lambda^{v}}{\text{min}}\;\sum_{v=1}^{V}\;\lambda^{v}\left(\right\|{\bar{X}}^{v\text{T}}-B^{v\text{T}}{S\|}_{F}^{2}+{\alpha \|S\|}_{F}^{2})+\sum_{v=1}^{V}{\left(\lambda^{v}\right)}^{\omega }, \end{array}$$ (6)

### 3.4 Graph contrastive regularization

Contrastive learning has gained popularity in unsupervised tasks. The fundamental concept of contrastive learning is to optimize the similarity between positive pairs while increasing the distance between negative pairs. In this study, each node and its K-nearest neighbors (KNN) are considered as positive pairs, and the contrastive regularization term is applied in Eq 6 for learning, resulting in the final consensus graph S. It can be represented as follows:

$$\begin{array}{c} \xi =\sum_{i=1}^{m}\sum_{j\in K_{i}^{v}}-lg\frac{\text{exp}(S_{ij})}{\sum_{p\neq i}^{N}\;\text{exp}\left(S_{ip}\right)}, \end{array}$$ (7)

Where $K_{i}^{v}$ denotes the K-nearest neighbors of node i in the v-th view. This regularization term is designed to enhance the similarity among positive pairs and diminish the similarity among negative pairs. Ultimately, our algorithm can be represented as follows:

$$\begin{array}{c} \underset{S,\lambda^{v}}{\text{min}}\;\sum_{v=1}^{V}\;\lambda^{v}\left(\right\|{\bar{X}}^{v\text{T}}-B^{v\text{T}}S{\|}_{F}^{2}+\alpha \xi )+\sum_{v=1}^{V}{\left(\lambda^{v}\right)}^{\omega }, \end{array}$$ (8)

### 3.5 Self-training clustering

Existing multi-view graph clustering algorithms mostly directly apply k-means or spectral clustering to the obtained consensus graph to acquire the final clustering labels. However, these algorithms suffer from significant randomness. Inspired by the DEC algorithm [28], we adopt self-training clustering to obtain the final labels, significantly improving the clustering stability.

We first define the normalized consensus graph $\hat{S}=W^{-1/2}S$, and matrix W is the diagonal matrix composed of the sums of each row in S. We conduct Singular Value Decomposition (SVD) on the matrix $\hat{S}{\hat{S}}^{\text{T}}$ to acquire the largest p singular values and their corresponding left and right singular vectors, denoted as *Y*Σ*L*^T^. Here, Σ = *diag*(*σ*_1_, ⋯, *σ*_*p*_) represents the singular values, while *Y* ∈ *R*^*m*×*p*^ are the left singular vectors and *L* ∈ *R*^*m*×*p*^ are the right singular vectors, respectively. We compute the final clustering matrix $C=\Sigma^{-\frac{1}{2}}Y^{\text{T}}\hat{S}$, and then perform self-training clustering on *C* = *C*^T^ [29].

We improve clustering iteratively by matching the target distribution through soft clustering. The clustering loss function is defined as follows:

$$\begin{array}{c} loss=KL\left(P\|Q\right)=\sum_{i}\;\sum_{j}\;p_{ij}\;\text{log}\frac{p_{ij}}{q_{ij}}, \end{array}$$ (9)

Where *KL*(⋅‖⋅) denotes the Kullback-Leibler divergence, Q represents the soft clustering labels, P represents the target distribution, and *q*_*ij*_ is the metric based on Student’s t-distribution [30]. It measures the resemblance among cluster *C*_*i*_ and cluster center *μ*_*i*_ and can be understood as the likelihood of allocating sample i to cluster j:

$$\begin{array}{c} q_{ij}=\frac{{\left(1+{\left\|C_{i}-\mu_{j}\right\|}^{2}\right)}^{-1}}{\Sigma_{j^{\prime }}{\left(1+{\left\|C_{i}-\mu_{j^{\prime }}\right\|}^{2}\right)}^{-1}}, \end{array}$$ (10)

In Eq 9, the target distribution P is obtained by squaring the term q and then normalizing it. It is defined as:

$$\begin{array}{c} p_{ij}=\frac{q_{ij}^{2}/\Sigma_{i}q_{ij}}{\sum_{j^{\prime }}q_{ij^{\prime }}^{2}/\Sigma_{i}q_{ij^{\prime }}}, \end{array}$$ (11)

Finally, our clustering labels are given by:

$$\begin{array}{c} label={argmax}_{j}q_{ij}, \end{array}$$ (12)

Where *q*_*ij*_ is calculated using Eq 10. If the change in labels of the target distribution between consecutive updates is less than the threshold *δ*, the training is terminated. We obtain the clustering results based on the previous iteration’s Q.

## 4 Optimization

In Eq 8, there are two sets of variables S and λ^*v*^. We utilize an alternating optimization approach, we keep one variable fixed while updating the other.

- Fix λ^*v*^, update S
  Treating λ^*v*^ as a constant, the optimization objective for S is as follows:
  $$\begin{array}{c} \underset{S}{\text{min}}\;\sum_{v=1}^{V}\;\lambda^{v}\left(\right\|{\bar{X}}^{v\text{T}}-B^{v\text{T}}S{\|}_{F}^{2}+\alpha \xi ), \end{array}$$ (13)
  We employ the gradient descent algorithm to solve for S, and the gradient of S can be decomposed into two parts. The first part is given by:
  $$\begin{array}{c} 2\sum_{v=1}^{V}\lambda^{v}\;(-{\left[B^{v}{\bar{X}}^{v\text{T}}\right]}_{ij}+{\left[B^{v}B^{v\text{T}}S^{\left(t-1\right)}\right]}_{ij}), \end{array}$$ (14)
  Let n be the total sum of the number of neighbors for all nodes, then the second term is:
  $$\begin{array}{c} \left\{ \begin{array}{c} \sum_{v=1}^{V}\lambda^{v}(-1+\frac{nexp(S_{ij}^{\left(t-1\right)})}{\sum_{p\neq i}^{m}\;\text{exp}(S_{ip}^{\left(t-1\right)})}),\;\text{if}\;j\in N_{i}^{v}\\ \sum_{v=1}^{V}\lambda^{v}(\frac{nexp(S_{ij}^{\left(t-1\right)})}{\sum_{p\neq i}^{m}\;\text{exp}(S_{ip}^{\left(t-1\right)})}),\;\text{other} \end{array},\right. \end{array}$$ (15)
  Then, we utilize the Adam optimization algorithm [31] to update S. To improve convergence speed, We get an initial *S*^+^ by Eq 6.
- Fix S, update λ^*v*^
  The loss function for λ^*v*^ is given by:
  $$\begin{array}{c} \underset{\lambda^{v}}{\text{min}}\;\sum_{v=1}^{V}\;\lambda^{v}\left(\right\|{\bar{X}}^{v\text{T}}-B^{v\text{T}}S{\|}_{F}^{2}+\alpha \xi )+\sum_{v=1}^{V}{\left(\lambda^{v}\right)}^{\omega }, \end{array}$$ (16)
  Setting its derivative to 0, we can obtain:
  $$\begin{array}{c} \lambda^{v}={\left(\frac{-\|{\bar{X}}^{v\text{T}}-B^{v\text{T}}{S\|}_{F}^{2}+\alpha \xi }{\omega }\right)}^{\frac{1}{\omega -1}}, \end{array}$$ (17)
  We alternately update S and λ^*v*^ until convergence. The entire process is outlined in Algorithm 1.

For the obtained similarity graph Z using Eq 4, we can directly perform spectral clustering to achieve the ultimate clustering result. Nevertheless, this algorithm has a time complexity of *O*(*N*^3^) and significant memory overhead, which is not suitable for scenarios with large datasets. Instead, we utilize node sampling algorithm to obtain a smaller similarity graph S using Eq 8 with a time complexity of only *O*(*m*^3^). Regarding the time cost of the gradient descent is *O*(*tVmn* + 2*tVm*), while generating the final clustering matrix Y and E is *O*(*m*^3^) and *O*(*m*^2^*N*) respectively, and the time cost of self-training clustering is *O*(*tm*^2^). In summary, our algorithm has higher efficiency compared to traditional graph learning algorithms.

**Algorithm 1**: SLMGC

**Data**: adjacency matrix $\tilde{A_{1}},\ldots ,\tilde{A_{V}}$, feature matrix *X*^1^, ⋯, *X*^*V*^, The graph filtering order k, and the parameters *α*, *ω*, *γ*, as well as the number of clusters p.

**Result**: Clustering label

**1** compute the normalized adjacency matrix $A_{v}={D_{v}}^{-\frac{1}{2}}\left(\tilde{A_{v}}+I\right){D_{v}}^{-\frac{1}{2}}$;

**2** compute the Laplacian matrix *L*_*v*_ = *I* − *A*_*v*_;

**3** perform graph filtering on each view’s feature matrix according to Eq 2;

**4** extract m samples and represent their indices as “ind.”;

**5** select m rows from the graph-filtered $\bar{X}$ using the indices “ind” to construct a new feature matrix B;

**6** **while** *not converged* **do**

**7**  Use the Adam optimization algorithm to update S;

**8**  **for** *each view* **do**

**9**   update λ^*v*^ using Eq 17.

**10**  **end**

**11** **end**

**12** compute the normalized consensus graph $\hat{S}$ and calculate the final clustering matrix C;

**13** Perform self-training clustering on *C*^T^;

**14** **while** *not converged* **do**

**15**  calculate the soft clustering labels Q and the target distribution P;

**16**  calculate the loss using Eq 9.

**17** **end**

**18** return Q;

**19** Obtain the final clustering labels using Eq 12.

## 5 Experiment

### 5.1 Dataset and evaluation metrics

We choose five datasets to assess our experiments. Among these datasets, ACM, DBLP, and IMDB consist of a feature matrix and several adjacency matrices. Amazon Photo and Amazon Computer [32] consist of multiple feature matrices and one adjacency matrix. The statistical information of the dataset statistics are presented in Table 1.

**Table 1:** Data set introduction.

Dataset	Nodes	Features	View and Edges	Clusters
ACM	3025	1830	Co-Author (2,210,761)	3
			Co-Subject (29,281)	
DBLP	4057	334	Co-Author (11,113)	4
			Co-Conference (5,000,495)	
			Co-Term (6,776,335)	
IMDB	4780	1232	Co-Actor (98,010)	3
			Co-Director (21,018)	
Amazon photos	7487	745	Co-Purchase (119,043)	8
			7487	
Amazon computers	13381	767	Co-Purchase (245,778)	10
			13381

- **ACM:** It is a paper network. We construct two views based on the co-paper (papers authored by the same authors) relationship and co-subject (papers with the same subjects) relationship. The paper features are represented as bag-of-words elements composed of keywords. We utilize the academic disciplines or subject areas of the papers as clustering labels;
- **DBLP:** It is an author network. It consists of three types of relationships: co-authorship (authors who have jointly authored papers), co-conference (authors who have published papers in the identical conferences), and co-term (authors who have utilized identical terminologies in their respective papers). The author features are represented as bag-of-words elements composed of keywords. We utilize the academic disciplines or subject areas of the authors as clustering labels;
- **IMDB:** This is a movie network. It employs two connections, co-actor (movies with common actors) and co-director (movies directed by common directors), to build a dual-view representation. The movie features are represented as bag-of-words elements composed of plots. We use movie genres as clustering label;
- **Amazon Photos and Amazon Computers:** They are portions of the Amazon co-purchase network dataset, where nodes correspond to products, and each product’s features are represented as bag-of-words from product comments. The relationships in the views are based on co-purchasing of products, and the clustering labels are product categories. To acquire multi-view attributes, the second feature matrix is formed by taking the Cartesian product.

We employ four commonly used metrics to showcase the effectiveness of our approach: Accuracy (ACC), Adjusted Rand Index (ARI), Normalized Mutual Information (NMI) and F1 score (F1).

### 5.2 Experimental setup and comparison models

The computer configuration used in this experiment is as follows: CPU is AMD Ryzen 5 4600H with Radeon Graphics, 6 cores and 12 threads, operating at 3.00GHz. The memory is 16GB at 3200MHz, and the GPU is NVIDIA GeForce GTX 1650 with 4GB of memory.

To validate our effectiveness, we compare SLMGC with several typical models. Among them, LINE and GAE are two traditional single-view clustering algorithm, and we average their results across each view to obtain the final metrics. PMNE is a multi-view clustering algorithm. RMSC is a robust multi-view spectral clustering algorithm utilizing Markov chains. PwMC and SwMC introduce weighted mechanisms for clustering multi-view data. O2MAC and O2MA are multi-view attribute graph clustering algorithms utilizing graph autoencoders. MAGCN is a multi-view attribute graph convolutional network, MAGC and MCGC are multi-view clustering algorithms that utilize graph learning modules.

For the Amazon Photo and Amazon Computer datasets, we will only compare with MAGCN, MAGC, and MCGC as they have shown superior performance compared to MGAE [33], ARVGAE [34], DAEGA [35], and GATE [36].

### 5.3 Experiment result

Tables 2 and 3 present the clustering results. In most measurements, our algorithm outperforms the reference algorithms on the ACM, DBLP, IMDB, Amazon Photo, and Amazon Computer datasets.

**Table 2:** Clustering results on ACM, DBLP, IMDB.

Algorithm	ACM				DBLP				IMDB			
	ACC	ARI	NMI	F1	ACC	ARI	NMI	F1	ACC	ARI	NMI	F1
GAE	0.7047	0.4409	0.4813	0.7088	0.5585	0.2618	0.3096	0.5475	0.4512	0.0483	0.0433	0.4285
LINE	0.6336	0.3402	0.3814	0.6495	0.8723	0.6966	0.6593	0.8532	0.4689	-0.009	0.0060	0.2858
PMNE	0.6901	0.4287	0.4601	0.6922	0.7917	0.5233	0.5880	0.7939	0.4907	0.0358	0.0357	0.3898
RMSC	0.6330	0.3354	0.4020	0.5776	0.9006	0.7673	0.7189	0.8310	0.2723	0.0019	0.0055	0.3781
PwMC	0.4157	0.0388	0.0301	0.3761	0.3220	0.0151	0.0180	0.2792	0.2447	0.0017	0.0022	0.3115
SwMC	0.3855	0.0188	0.0849	0.4737	0.6571	0.3832	0.3771	0.5644	0.2691	0.0004	0.0057	0.3747
O2MA	0.8880	0.6987	0.6515	0.8894	0.9040	0.7705	0.7257	0.8976	0.4697	0.0753	0.0524	0.4229
O2MAC	0.9042	0.7394	0.6923	0.9053	0.9074	0.7780	0.7287	0.9013	0.4502	0.0564	0.0421	0.4159
MAGC	0.8806	0.6808	0.6180	0.8835	0.9282	0.8267	0.7768	0.9237	0.6125	0.1806	0.1167	0.4551
MCGC	0.9147	0.7627	0.7126	0.9155	0.9298	0.7746	0.8302	0.9252	0.6182	0.1833	0.1149	0.4401
SLMGC	0.9372	0.8317	0.8218	**0.9344**	0.9320	0.8351	0.7808	0.9210	0.5718	0.0845	0.0389	0.3962

**Table 3:** Clustering results on Amazon Photo and Amazon Computer.

Algorithm	Feature Matrix	Amazon Photo				Amazon Computer			
		ACC	ARI	NMI	F1	ACC	ARI	NMI	F1
MAGCN-view1	X1	0.3775	0.3321	0.2413	0.3320	0.3357	0.1185	0.1666	0.3493
MAGCN-view2	X2	0.3019	0.0480	0.1313	0.3279	∼	∼	∼	∼
MAGC-view1	X1	0.4410	0.1060	0.4192	0.3282	0.5730	0.2198	0.4376	0.4703
MAGC-view2	X2	0.3844	0.0605	0.3637	0.2788	0.5727	0.2194	0.4366	0.4698
MCGC-view1	X1	0.6903	0.4244	0.6166	0.6735	0.5851	0.3811	0.5252	0.5238
MCGC-view2	X2	0.7103	0.4396	0.6048	0.6764	0.5889	0.3896	0.5244	0.5101
SLMGC-view1	X1	0.5783	0.3710	0.4473	0.5216	0.4582	0.2462	0.3828	0.4628
SLMGC-view2	X2	0.5669	0.3253	0.4239	0.5217	0.4299	0.2273	0.3101	0.3281
MAGCN-multi-view	X1,X2	0.4835	0.2105	0.3550	0.4416	∼	∼	∼	∼
MAGC-multi-view	X1,X2	0.4511	0.1127	0.4297	0.3359	0.6080	0.2958	0.4395	0.5080
MCGC-multi-view	X1,X2	0.7164	0.4323	0.6154	0.6864	0.5967	0.3902	0.5317	0.5204
SLMGC-multi-view	X1,X2	0.5802	0.3013	0.4330	0.5478	0.4733	0.2714	0.3575	0.3126

The ‘∼’ indicates that the algorithm encounters an out-of-memory problem.

### 5.4 Run time comparison

Next, we compared the runtime of SLMGC with two top-performing deep neural network algorithms, O2MAC and MAGCN, as well as graph learning algorithms MAGC and MCGC on five datasets. The specific performance is presented in Table 4.

**Table 4:** Run time comparison. (seconds).

Algorithm	ACM	DBLP	IMDB	Amazon Photo	Amazon Computer
O2MAC	2079.43	6059.71	7930.58	∼	∼
MAGCN	∼	∼	∼	5947.31	∼
MAGC	189.72	203.11	247.40	1859.13	5331.09
MCGC	249.18	301.17	359.75	2688.83	6595.71
SLMGC	114.32	137.47	153.83	1427.22	4738.60

The ‘∼’ indicates that the algorithm encounters an out-of-memory problem.

### 5.5 Ablation study

In this part, we conducted a series of experiments to investigate the impact of each parameter in the algorithm, comprising the order of graph filtering k, the number of sampled points m and its parameter *γ* for sample extraction, as well as the parameters *α* and *ω* in graph contrastive learning.

**Fig 2:**
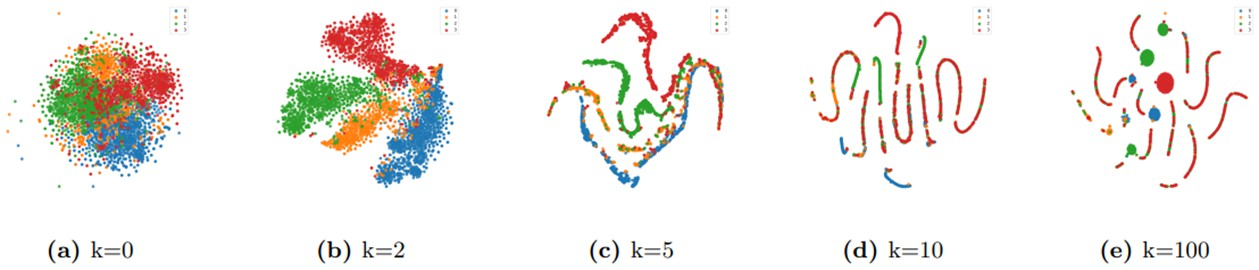
The t-SNE visualization of node features in DBLP dataset under different filter orders (k = 0, 2, 5, 10, 100).

In theory, the higher the order of graph filtering, the better it can capture global information. Therefore, we set k=[0, 2, 5, 10, 100] and performed t-SNE visualization on node features under different filter orders on the DBLP dataset, as shown in Fig 2.

It can be observed that graph filtering indeed processes the node features effectively. However, a higher filter order does not necessarily lead to better results; excessively high filter orders can make the node features too similar, making it difficult to distinguish between them. Through experiments, we have found that a filter order of 2 is a preferable choice.

In order to assess the efficacy of graph filtering, we performed comparative experiments on three datasets: ACM, DBLP, and IMDB. We compared the results obtained without using graph filtering and with using 2nd-order graph filtering. It can be observed that graph filtering indeed improves the final clustering results, demonstrating the effectiveness of graph filtering. The results are presented in Table 5.

**Table 5:** The effectiveness of graph filtering on ACM, DBLP, and IMDB.

Dataset	Graph Filtering	ACC	ARI	NMI	F1
ACM	0	0.9017	0.7885	0.7248	0.8962
	2	0.9372	0.8317	0.8218	0.9344
DBLP	0	0.9147	0.7985	0.7348	0.9062
	2	0.9320	0.8351	0.7808	0.9280
IMDB	0	0.5447	0.0432	0.0308	0.3351
	2	0.5718	0.0845	0.0389	0.3962

**Fig 3:**
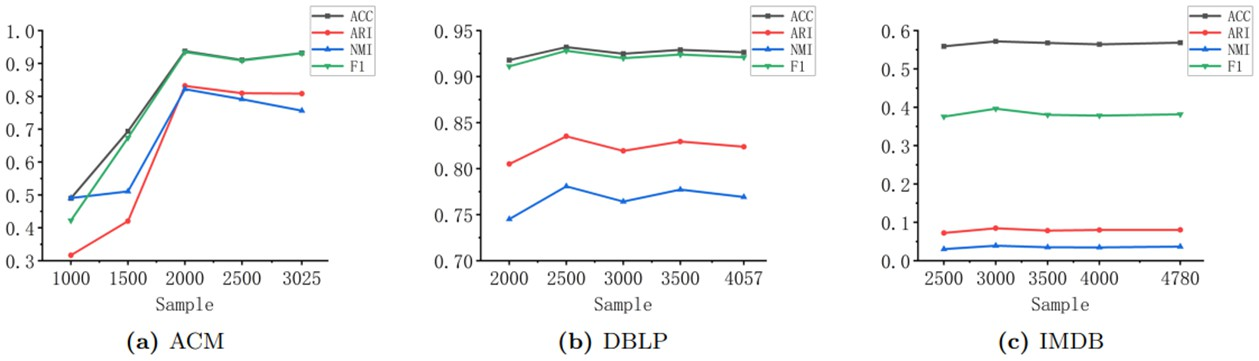
The performance of sample extraction size in ACM, DBLP, and IMDB datasets.

In the previous sections, we proposed the theory of sample extraction, which can reduce computation time, save memory overhead, and avoid the influence of outliers on the final clustering. Next, we conduct experiments and analysis on the sample extraction size in the ACM, DBLP, and IMDB datasets. We start by sampling every 500 nodes downwards from the total number of original nodes. It is observed that too few sample points can result in insufficient information, affecting the final clustering metrics. Through experiments, we found that sample extraction size around 2/3 of the total number of nodes yields the best performance. See Fig 3.

**Fig 4:**
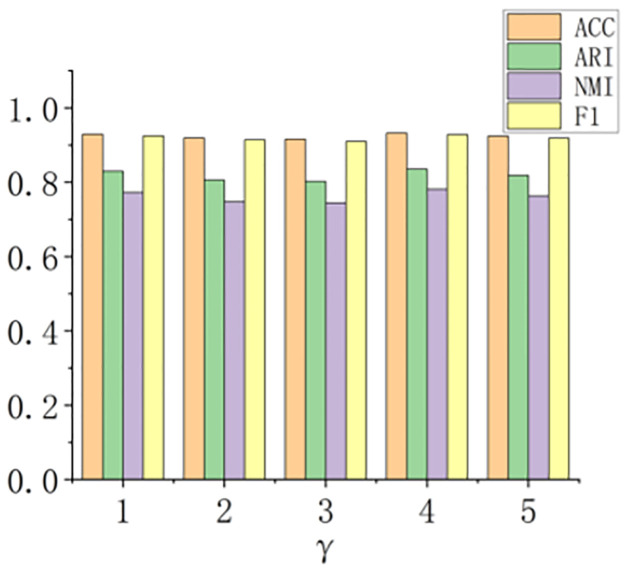
The impact of the sample extraction parameter *γ* in DBLP.

The parameter *γ* has a very small impact on sample extraction, as shown in Fig 4. In our experiments, we use a slightly superior value of 4 for *γ*.

**Fig 5:**
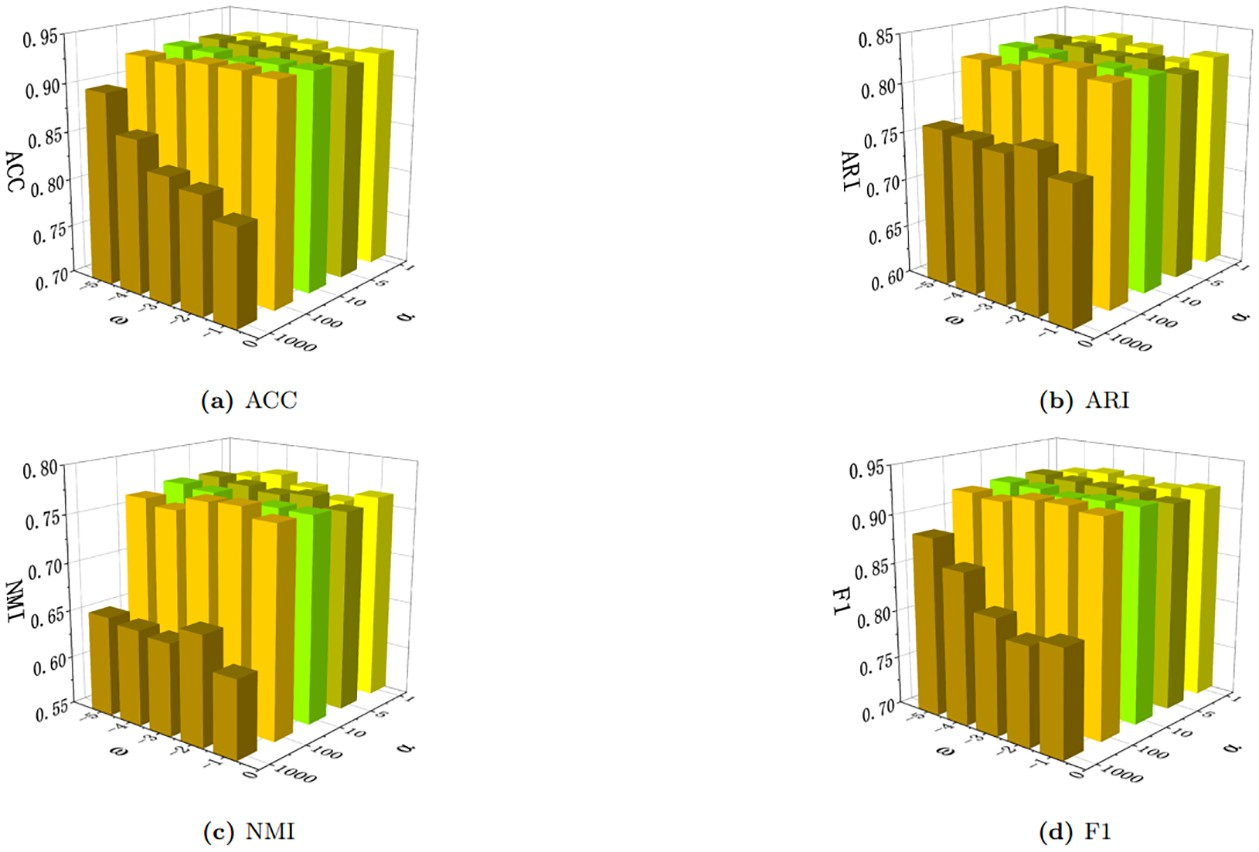
Sensitivity analysis of DBLP to parameters *α* and *ω*.

Finally, we conduct experiments on the two parameters in graph contrastive learning. We set *α*=[1, 5, 10, 100, 1000] and *ω*=[-1,-2,-3,-4,-5], and observe their effects on the four evaluation metrics on the DBLP dataset. As shown in Fig 5, our algorithm performs less favorably under higher *α* values, but it is not sensitive to lower *α* values and *ω*. This demonstrates the robustness of our algorithm within a certain range of these parameters, making it applicable and meaningful in practical scenarios.

### 5.6 Discussion

Through experiments on the ACM, DBLP, and IMDB datasets, we found that O2MA, MAGC, MCGC, and our SLMGC algorithm outperform single-view algorithms GAE and LINE. This is because single-view algorithms cannot leverage multiple-view relationships, demonstrating the superiority of multi-view clustering. However, early multi-view clustering methods such as PMNE, RMSC, PwMC, and SwMC show poor performance as they cannot simultaneously utilize node attributes and structural relationships.

It can be observed that the three algorithms using graph learning modules perform better than several methods using deep neural networks. This is attributed to the fact that graph learning modules can simultaneously leverage node attributes and structural relationships while fully considering information from all views. In the case of our method within the graph learning module algorithm, it does not perform as well as MAGC and MCGC on the IMDB dataset. This is because our node sampling method, in datasets with numerous nodes, discards a significant number of nodes, leading to a loss of accuracy.

On the Amazon Photo and Amazon Computer datasets, our SLMGC algorithm exhibits a significant advantage compared to the deep neural network algorithm MAGCN. This is because our algorithm considers not only various node attributes but also the structural relationships in the graph. However, our algorithm has a considerable disadvantage compared to the other two graph learning module algorithms on the Amazon Computer dataset. This is because, in samples with multiple node attributes, discarding more nodes results in greater loss.

Although our algorithm sacrifices some accuracy through sampling, it significantly outperforms in terms of computational time. The comparison of runtime indicates that graph learning module algorithms have much shorter runtimes compared to deep neural network algorithms. Additionally, our algorithm saves 40% more time than MAGC.

## 6 Conclusions

Existing graph clustering algorithms heavily rely on deep learning networks, and multi-view clustering is still in its early stage with many unresolved issues. This paper proposes a sampling-based graph learning multi-view clustering algorithm. We introduce graph filtering to reduce noise in the original graphs, followed by extracting a subset of node samples based on their importance. This approach reduces computational cost while maintaining clustering accuracy. Moreover, we incorporate a graph contrastive regularization term to enhance the graph learning module. Finally, we employ self-training clustering to reduce potential errors in traditional clustering algorithms. We compare our algorithm with popular and well-performing deep learning graph clustering algorithms on five datasets, and the experimental results demonstrate the superiority of our proposed approach.

## Supporting information

S1 Code SLMGC. (ZIP)

S1 Dataset (ZIP)
